# Circadian Rhythm Gene PER3 Negatively Regulates Stemness of Prostate Cancer Stem Cells via WNT/β-Catenin Signaling in Tumor Microenvironment

**DOI:** 10.3389/fcell.2021.656981

**Published:** 2021-03-18

**Authors:** Qilin Li, Ding Xia, Zhihua Wang, Bo Liu, Jing Zhang, Ping Peng, Qiujun Tang, Jie Dong, Juan Guo, Dong Kuang, Weimin Chen, Jing Mao, Qiuhui Li, Xin Chen

**Affiliations:** ^1^Department of Stomatology, Tongji Hospital, Tongji Medical College, Huazhong University of Science and Technology, Wuhan, China; ^2^Department of Urology, Tongji Hospital, Tongji Medical College, Huazhong University of Science and Technology, Wuhan, China; ^3^Department of Oncology, Tongji Hospital, Tongji Medical College, Huazhong University of Science and Technology, Wuhan, China; ^4^Department of Pathology, Tongji Hospital, Tongji Medical College, Huazhong University of Science and Technology, Wuhan, China; ^5^State Key Laboratory Breeding Base of Basic Science of Stomatology (Hubei-MOST) and Key Laboratory for Oral Biomedicine of Ministry of Education (KLOBM), School and Hospital of Stomatology, Wuhan University, Wuhan, China

**Keywords:** prostate cancer stem cells, prostate cancer, tumor microenvironment, PER3, Wnt/β-catenin signaling

## Abstract

Prostate cancer (PCa) cells are heterogeneous, containing a variety of cancer cells with phenotypical and functional discrepancies in the tumor microenvironment, where prostate cancer stem cells (PCSCs) play a vital role in PCa development. Our earlier studies have shown that ALDH^hi^CD44^+^ (DP) PCa cells and the corresponding ALDH^lo^CD44^–^ (DN) PCa cells manifest as PCSCs and non-PCSCs, respectively, but the underlying mechanisms regulating stemness of the PCSCs are not completely understood. To tackle this issue, we have performed RNA-Sequencing and bioinformatic analysis in DP (versus DN) cells in this study. We discovered that, PER3 (period circadian regulator 3), a circadian rhythm gene, is significantly downregulated in DP cells. Overexpression of PER3 in DP cells significantly suppressed their sphere- and colony-forming abilities as well as tumorigenicity in immunodeficient hosts. In contrast, knockdown of PER3 in DN cells dramatically promoted their colony-forming and tumor-initiating capacities. Clinically, PER3 is downregulated in human prostate cancer specimens and PER3 expression levels are highly correlated with the prognosis of the PCa patient. Mechanistically, we observed that low levels of PER3 stimulates the expression of BMAL1, leading to the phosphorylation of β-catenin and the activation of the WNT/β-catenin pathway. Together, our results indicate that PER3 negatively regulates stemness of PCSCs via WNT/β-catenin signaling in the tumor microenvironment, providing a novel strategy to treat PCa patients.

## Introduction

Human prostate cancer (PCa) is the most malignant cancers affecting men worldwide with an increasing incidence and high mortality. It has been estimated that there were 191,930 new cases and 33,330 deaths from PCa in the United States during 2020 (Siegel et al., 2020). Surgery and/or radiation are often curative in the initial stages, but many patients eventually develop metastatic castration resistant prostate cancer (mCRPC) that is incurable and fatal (Rycaj and Tang, 2017; Li et al., 2018). At the moment, the etiology for PCa development is still not completely understood, and novel regiments for treating PCa metastasis and recurrence still need to be developed.

Prostate cancer is a heterogenous malignancy, consisting of cancer cells with functional and phenotypical differences in the tumor microenvironment (TME) (Skvortsov et al., 2018). Tumor cell heterogeneity results from clone evolution and/or cancer stem cell (CSC) models (Tang, 2012; Prager et al., 2019). Like many other solid tumors, emerging evidence has shown that there are subsets of PCa cells with stem cell properties in TME, i.e., prostate cancer stem cells (PCSCs), responsible for PCa initiation, progression, therapy resistance and metastasis (Chen et al., 2013; Skvortsov et al., 2018). Hence, a better understanding of PCSCs in TME will result in better treatment for PCa patients. On the other hand, TME is vital for PCSCs. Recent studies have suggested that certain signaling pathways in TME may play a critical role in regulating PCSCs for their SC properties, metastatic traits and resistance to treatment, including the CXCL12/CXCR4 and WNT/β-catenin signaling pathways (Trautmann et al., 2014; Cojoc et al., 2015). However, it is not completely clear which molecules are required to activate these signaling pathways that regulate PCSCs in TME.

The circadian clock is a molecular pacemaker and an evolutionally conserved mechanism that governs biological and physiological processes, which is located in the hypothalamus suprachiasmatic nucleus (Shafi and Knudsen, 2019). The circadian rhythm is generated by positive and negative transcription-translation feedback loops (Young and Kay, 2001). In general, the positive loop is composed of the basic helix-loop-helix heterodimeric transcriptional factors, BMAL1 and CLOCK, which drive the clock and are highly associated with the regulation of the immune response and various cellular pathways (Dong et al., 2019). In addition, the BMAL1/CLOCK complex regulate the expression of the negative regulators of the loop, including *Period* (*PER1*, *PER2*, *PER3*) and *Cryptochromes* (*CRY1* and *CRY2*) gene, which in turn repress BMAL1/CLOCK activity (Shafi and Knudsen, 2019). Recent evidence has reported that circadian rhythm disruption is related to increased cancer incidence (Dickerman et al., 2016; Markt et al., 2016; Wendeu-Foyet and Menegaux, 2017) and poor efficacy of cancer management (Sancar et al., 2015), although the pertinent mechanisms are incompletely understood. Importantly, very recent studies have suggested that uncovering the underlying mechanisms of circadian clock regulation in cancer development may explore a future direction in cancer treatment (Sancar and Van Gelder, 2021).

Whether circadian rhythm genes regulate stemness of CSCs is incompletely understood, and dysregulation of circadian clock in CSCs are correlated to cancer development. For instance, Puram et al. (2016) have indicated that altered circadian regulation in leukemia stem cells may be associated with cancer progression. In solid tumors, targeting CSCs in brain tumors via disruption of the circadian clock genes may be a novel strategy for targeted therapy of glioblastoma patients (Dong et al., 2019). Based on this background, we started this project to understand the molecular impact of circadian regulation in PCSCs. In this study, we performed a deep RNA-sequencing to compare gene expression profiles, using ALDH^hi^CD44^+^ (DP) PCa cells and ALDH^lo^CD44^–^ (DN) PCa cells, which have been shown to function as PCSCs and non-PCSCs, respectively (Li et al., unpublished). We found that: (1) PER3 (Period Circadian Regulator 3), a circadian rhythm gene, is markedly downregulated in DP PCa cells. (2). PER3 overexpression in DP PCa cells significantly inhibits their clonogenicity and tumorigenicity, whereas PER3 knockdown in DN cells dramatically promotes their colony-forming and tumor-initiating abilities. (3) PER3 is downregulated in human PCa specimens, and its level is related with better patient survival. (4) Mechanistically, low expression levels of PER3 stimulates the expression of BMAL1, resulting in the phosphorylation of β-catenin and the activation of the WNT/β-catenin pathway. Collectively, this study identifies that PER3 is a negative regulator for PCSCs via the activation of WNT/β-catenin signaling in TME, providing a potential and novel strategy for PCa treatment.

## Materials and Methods

### Cell Lines and Reagents

Human prostate cancer cell lines, PC3 and DU145, were purchased and authenticated from Cell Bank (Chinese Academy of Sciences, Shanghai, China). Cells were cultured in DMEM (Invitrogen, CA, United States) with 10% FBS (Gibco, United States) and 10,000 U/ml penicillin, and incubated in 5% CO2 incubator at 37°C. All cells were tested for mycoplasma free via a mycoplasma detection kit (Thermo Fisher Scientific, United States).

### Purification of Double Marker Cells by Fluorescence-Activated Cell Sorting (FACS)

Basic procedures were previously described (Chen et al., 2016). In brief, all cells were first digested into single cells by trypsin containing 0.25% EDTA, and resuspended in PBS. Cells were incubated with a PE-conjugated anti-CD44 antibody (cat#: 550392, BD Pharmingen) at 4°C for 30 min, and then incubated with an ALDEFLUOR assay kit (Stemcell Technologies, #01700) at 37°C for 45 min, according to the manufacturer’s instructions. The top 10% and bottom 10% gated cells were resolved and debris was abducted by gates in the light scatter SSC versus FSC diagrams.

DP PCa cells and the corresponding DN PCa cells were sorted by a FACS sorter (Aria II, BD Biosciences, United States).

### Sphere and Colony-Formation Assays

Basic procedures were previously described (Chen et al., 2016). For sphere-formation assay, purified PC3 DP and DN cells were seeded (1,000 cells/well) in 96-well ultra-low attachment plates (Corning) and incubated using DMEM/F12 (Invitrogen, United States) supplemented with B27 (GIBCO) and bFGF (Stem Cell Technology). For the sphere passage, spheres were collected with a 40 μm diameter cell strainer, and then digested with 0.25% EDTA trypsin for 15 min into single cells for the next cycle. Spheres (diameter >40 μm) were counted, photographed and scored under an inverted microscope (Olympus CKX41, Tokyo, Japan) to determine the sphere forming efficiency. For the colony-formation assay, purified PC3 DP and DN cells were resuspended in Matrigel (BD Biosciences, Cat#: 354256) and seeded into 24-well culture plates (1,000 cells/well). After 30 min at 37°C, colony-formation culture medium was added to the wells: 50 ng/ml recombinant human EGF (Sigma), 10 nM Gastrin (Sigma), DMEM/F12 (Invitrogen, United States) supplemented with 1× B27 (GIBCO) and 500 nM A83-01 (Miltenyi Biotec). To determine the colony-forming efficiency, colonies (diameters >100 μm) were numerated, photographed and scored after 1 week.

### Animal Studies

NOD/SCID male mice (4-week-old) were purchased from Beijing HFK Bioscience Co., Ltd (Beijing, China). Our animal work was conducted under the protocols approved by the IACUC of Huazhong University of Science and Technology (HUST; Wuhan, China, IACUC No. S2348). Basic procedures have been previously described (Chen et al., 2016). For limiting-dilution tumor regeneration assays (LDA), cells at increasing doses (100 to 1,000 cells/injection) were injected subcutaneously into NOD/SCID male mice. At the endpoint, mice were anesthetized and sacrificed according to AVMA guidelines, and tumors were harvested. The tumor-initiating frequency (TIF) in this study was calculated^1^.

### RT-qPCR

Basic procedures were previously described (Chen et al., 2016). Total RNA from PC3 DP and DN cells was extracted using the RNAiso Plus (Takara, Japan, Cat#: 9108), and cDNA was synthesized using cDNA Synthesis Kit (Thermo Scientific, United States, Cat#: 11754050). RT-qPCR analysis was performed using Maxima SYBR Green/ROX Mix (Thermo Scientific, United States) according to the manufacturer’s instructions. Glyceraldehyde-3-phosphate dehydrogenase (GAPDH) was used as an internal control. GAPDH: 5′-TCGTGGAAGGACTCATGACC-3′ (forward) and 5′TCC ACCACCCTGTTGCTGTA-3′ (reverse); CD44: 5′-ATGG ACAAGTTTTGGTGGCACGC-3′ (forward) and 5′-AAGATGT AACCTCCTGAAGTGCTGC-3′ (reverse); ALDH1A1: 5′-ATCT TTGCTGACTGTGACCT-3′ (forward) and 5′-GCACCTCTT xCTACCACTCTC-3′ (reverse); PER3: 5′-AGCTACCTGCACC CTGAAGA-3′ (forward) and 5′-CGAACTTTATGCCGACC AAT-3′ (reverse); CD133: 5′-TGGATGCAGAACTTGACAA CGT-3′ (forward) and 5′-ATACCTGCTACGACAGTCGTGGT-3′ (reverse).

### Immunofluorescence

Procedures for immunofluorescent staining was carried out as previously described (Chen et al., 2016). In brief, purified PC3 DP and DN cells were cultured in glass-bottomed 24-well dishes overnight, and incubated with primary antibodies (anti-PER3, Abcam, Cat#: ab67862, 1:100, or anti-β-catenin, Cell Signaling Technology, Cat#2951, 1:100) followed by secondary antibodies conjugated to Streptavidin-Cy3 (1:100; Thermo Fisher Scientific, Cat#:A12421) or Alexa Fluor 488 (1:100; Invitrogen, Cat#:A21202). Finally, cellular nuclei were stained with DAPI dye (4′,6-dynamiting-2-phenylamine, Sigma, Cat#: D9542) at room temperature for 10 min. Images were captured under a fluorescence microscope (Olympus, Tokyo, Japan).

### Western Blotting

Procedures for western blotting have been previously described (Li et al., 2018). Primary antibodies used for western blotting are as follows: anti-GAPDH (Abcam, Cat#: ab9484, 1:5,000), anti-CD44 (Abcam, Cat#: ab157107, 1:5,000), anti-PER3 (Abcam, Cat#: ab177482, 1:5,000), anti-ALDH1 (Cell Signaling Technology, Cat#:5741, 1:5,000), anti-total β-catenin (Cell Signaling Technology, Cat#:2951, 1:5,000), anti-phosphorylated β-catenin (Cell Signaling Technology, Cat#: 176, 1:5,000).

### Gene Overexpression and Knockdown With Lentiviral Vectors

Basic procedures were previously described (Chen et al., 2016). Briefly, PER3-shRNA lentivirus, pGMLV-PE1-PER3 lentiviral vectors were purchased from Shanghai SBO Medical Biotechnology (Shanghai, China). Purified PC3 DP and DN cells were purified via FACS and seeded in 6-well plates (50, 000 cells/well) and cultured for 24 h. PC3 DP were infected with pGMLV-PE1-PER3 lentiviral vectors, and PC3 DN cells were infected with PER3 shRNA-encoding viral vectors, at a multiplicity of infection (MOI) of 20 for 48 h, respectively.

### Evaluation of WNT Activity

A TCF/LEF lentiviral reporter, tagged the gene expressing GFP (termed as TOP-GFP), has been widely used for evaluating WNT activity (Vermeulen et al., 2010). Lentivirus generating from the TCF/LEF reporter was purchased from Shanghai SBO Medical Biotechnology (Shanghai, China), and used for cell infection at the MOI of 20 for 48 h. To evaluate WNT activity, the intensity of GFP were detected via a fluorescent imaging system (Olympus Corp.).

### RNA-Sequencing (RNA-seq)

Procedures for RNA-Seq have been previously described (Li et al., 2018). FACS was used to purify PC3 DP and DN cells, and the total RNA was extracted (RNeasy mini kit, Qiagen). A 1 μg of RNA was used for cDNA libraries per manufacturer’s instructions (NEBNext^®^ UltraTM RNA Library Prep Kit for Illumina^®^, NEB, United States). TruSeq PE Cluster Kit v3-cBot-HS (Illumia) was used for Clustering of the index-coded samples, and the library preparations were then sequenced on an Illumina NovaSeq platform with 150 bp paired-end reads generated. Gene model annotation files and reference genome were downloaded^2^. Hisat 2 v2.0.5 was used to build an index of the reference genome. Cluster 3.0 and Java Tree View were used for Heatmaps.

### Bioinformatic Analysis

Basic procedures were previously described (Li et al., 2018). KEGG was used to analyze pathways that are preferentially enriched in PC3 DP or DN cells. Gene Set Enrichment Analysis (GSEA) was performed at version 4.0.3. The software was run per the user’s guide. An FDR < 0.25 is considered as statistically significant.

### Tissue Microarray (TMA) and Immunohistochemical Staining (IHC)

Basic procedures were previously described (Li et al., 2018). We purchased tissue microarrays (TMA) from http://www.avilabio.com (Shanxi, China), which consist of 16 normal/benign prostatic tissues and 32 prostate tumor cores. IHC for PER3 (Abcam, #ab67862, 1:100) staining was conducted and PER3 scoring was calculated by two experienced pathologists via an Image-pro Plus 6.0 system (Media Cybernetics, Inc., United States).

### Statistical Analysis

In this study, the unpaired two-tailed Student’s *t*-test was used to compare significance in sphere- and colony-forming efficiencies, tumor weight, and knockdown efficiency. Survival curve was generated from TCGA database. At least three independent experiments were performed for mean standard deviation. *P* < 0.05 was considered statistically significant.

### Data Availability

The raw RNA-seq data is deposited in Sequence Read Archive (SRA) database^3^ (accession number: PRJNA671757).

## Results

### PC3 Double-Positive (ALDH^hi^CD44^+^) Prostate Cancer Cells Bear PCSC Properties in TME

Emerging evidence has shown that PCSCs are enriched by different phenotypic markers, including expression of CD44^+^, aldehyde dehydrogenase (ALDH), CD44^+^α2β1^hi^CD133^+^, PSA^–/lo^, and CD166^+^ (Chen et al., 2013; Skvortsov et al., 2018). Using HPCa treatment-naïve samples, we reported earlier that FACS-purified ALDH^hi^CD44^+^ PCa cells (double-positive/DP) seem to have higher colony-forming abilities than the isogenic ALDH^lo^CD44^–^ (double-negative/DN) cells in androgen-deprived cultured conditions (Chen et al., 2016), suggesting that ALDH^hi^CD44^+^ PCa cells may enrich for PCSCs in TME.

**FIGURE 1:**
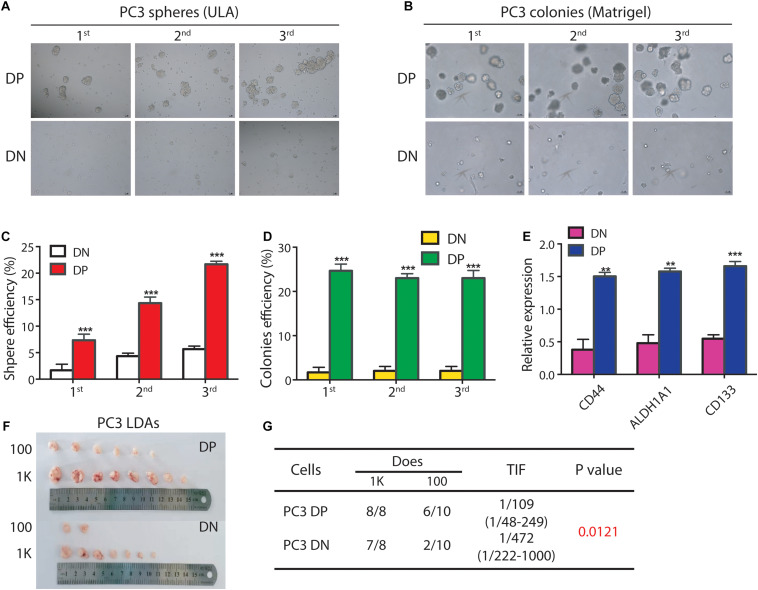
The PC3 ALDH^hi^CD44^+^ PCa cells enrich for PCSCs. **(A,B)** The PC3 ALDH^hi^CD44^+^ (DP) PCa cells have higher clonogenicity than isogenic ALDH^lo^CD44^–^ (DN) PCa cells. **(A)** For sphere assays, PC3 DP and corresponding DN cells were freshly purified by FACS and seeded in ultra-low attachment (ULA) plates (1,000 cells/well). Spheres were counted and photographed 12 days later. The 1° spheres were harvested and passaged for 2° spheres (1,000 cells/well) in ULA plates, which were then harvested for 3° spheres. **(B)**. For colony formation assays, PC3 DP and DN cells were mixed with Matrigel (1,000 cells/well) in 24-well cell culture plates. The 1° colonies were harvested and passaged for 2° colony-formation assays (1,000 cells/well), which were then harvested for 3° clonogenic assays. Data is presented from three separate experiments. Scale bars, 100 μm. **(C)** Spheres were counted for sphere formation assays **(A)** and the sphere efficiency was presented for three generations. ***P < 0.001. **(D)** Colonies were enumerated for colony-formation assays **(B)** and the colony efficiency was shown for 3 generations. ***P < 0.001. **(E)** Expression of mRNA levels for *CD44*, *ALDH1A1*, and *CD133* is much higher in PC3 DP cells (vs. DN cells). GAPDH was served as a loading control. Data was collected from there independent experiments. **P < 0.01, ***P < 0.001. **(F,G)** PC3 DP cells are more tumorigenic than PC3 DN cells in male NOD/SCID mice. PC3 DP and DN cells were freshly sorted via FACS, and injected subcutaneously in male NOD/SCID mice for limiting dilution assays (LDAs). Six weeks after implanting, tumors were harvested. Tumor images, incidence and tumor-initiating frequency (TIF) were recorded. TIF was calculated using Extreme Limiting Dilution Analysis (ELDA) software (http://bioinf.wehi.edu.au/software/elda/index.html).

To validate this suggestion, we used FACS to purify DP and corresponding DN cells in the PC3 cell line, and tested their sphere- and colony-forming abilities. We found that PC3 DP cells have a higher sphere-forming capacity compared to DN cells (Figures 1A,C). For example, in the 1° generation, PC3 DP cells demonstrated ∼4.7-fold higher sphere-forming ability compared to PC3 DN cells (Figures 1A,C) in ultra-low attachment plate (ULA). In the 2° generation, PC3 DP cells generated bigger and more spheres than PC3 DN cells (Figures 1A,C), and this trend continued to the 3° generation (Figures 1A,C). Moreover, PC3 DP cells exhibited higher clonogenic activities than the DN cells for three consecutive generations by generating more and larger colonies in Matrigel (Figures 1B,D). Furthermore, we purified PC3 DP and DN cells and performed RT-qPCR analysis of the stem cell associated genes. This analysis revealed that PC3 DP cells expressed higher levels of *CD44*, *ALDH1A1*, and *CD133* mRNA levels (Figure 1E). Thus, PC3 DP cells bear CSC features *in vitro*.

As *in vivo* limiting-dilution tumor regeneration assay (LDA) is widely accepted as the standard strategy for examining the tumor-initiating frequency in a candidate CSC population (Chen et al., 2013), we freshly sorted PC3 DP and isogenic DN cells and subcutaneously (s.c.) injected these cells in male NOD/SCID mice at different doses (from 100 to 1,000; Figures 1F,G). Expectedly, as few as 100 PC3 DP cells generated 6/10 tumors, while 100 DN cells gave rise to 2/10 tumors (Figures 1F,G). Overall, PC3 DP cells have ∼4-fold higher tumor-initiating ability than DN cells (TIF 1/109 vs. 1/472, respectively, *P* = 0.0121, Figure 1G). Together, those results showed that PC3 DP cells bear PCSC characteristics.

### RNA-Seq Identifies PER3 Prominently Downregulated in PC3 DP Cells

**FIGURE 2:**
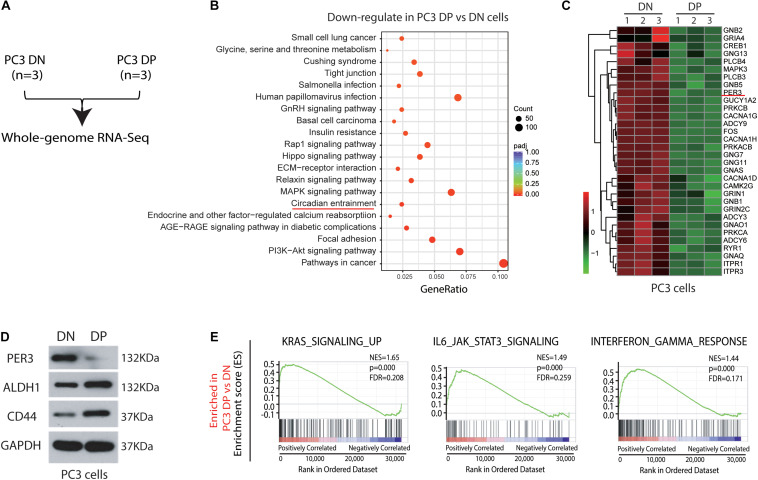
RNA-Sequencing and bioinformatic analysis identify circadian rhythm genes as potential regulators for PCSCs in TME. **(A)** Experimental plan for RNA-Seq in PC3 cell model. **(B)** KEGG enrichment of 4,672 DEGs that were downregulated in PC3 DP cells (vs. PC3 DP cells) (*P* < 0.05; | log_2_Fold Change| > 0) identified 20 significantly down-regulated pathways. In particular, the circadian entrainment pathway was marked in red. **(C)** Heatmaps showing representative circadian rhythm genes downregulated in PC3 DP cells compared to DN cells. **(D)** PER3 level is downregulated in PC3 DP cells at the protein level, whereas CD44 and ALDH1 are upregulated in PC3 DP cells (vs. DN cells). **(E)** GSEA showing that PC3 DP cells are enriched in gene sets preferentially expressed in stem cell pathways, including KRAS signaling, and IL6/JAK/STAT3 signaling, and also in interferon responses. Data are presented from three independent experiments.

Previous studies have proposed that PCSCs play vital roles in TME (Skvortsov et al., 2018), but the exact regulatory mechanisms are not completely understood. To tackle this issue, we ran an RNA-Sequencing (RNA-Seq) assay using freshly purified PC3 DP and corresponding DN cells (Figure 2A; Supplementary Figure 1). RNA-Seq studies found that 5,053 differentially expressed genes (DEGs) are upregulated in DP cells, whereas 4,672 DEGs are downregulated in DP cells (*P* < 0.05; |log_2_Fold Change| > 0). Moreover, bioinformatic analysis using KEGG revealed novel signaling pathways significantly downregulated in PC3 DP cells (Figure 2B). Surprisingly, KEGG demonstrated that the circadian entrainment pathway is among the notably downregulated pathways, as recent evidence has suggested that circadian rhythm genes have potential roles in TME (Zhou et al., 2020; Figure 2B). Remarkably, PER3 (Period Circadian Regulator 3), a circadian rhythm gene, which seems to play a vital role in other solid tumors (Wang et al., 2012), is significantly downregulated in PC3 DP cells (Figure 2C). With a fold change (FC) ≥ 2 and a *P* value < 0.05, we found that a total of 2,693 DEGs were identified between PC3 DP and DN cells, and PER3 was still among the most downregulated DEGs in DP cells (Supplementary Table 1). In support, PC3 DP cells have lower levels of PER3 than isogenic DN cells at the protein level, whereas CSC markers (ALDH1 and CD44) are significantly upregulated in PC3 DP cells (Figure 2D). Furthermore, GSEA (Gene Set Enrichment Analysis) was applied to explore the signaling pathways associated with TME, and the results revealed that PC3 DP cells are enriched in stem cell pathways, including KRAS signaling, JAK/STAT3 signaling, and STAT5 signaling (Figure 2E; Supplementary Figure 2A). Additionally, PC3 DP cells showed an increased inflammatory and interferon response (Figure 2E; Supplementary Figure 2B). Combined, these data suggest that PER3 is a potentially important factor for regulating PCSCs in TME.

### PER3 Negatively Regulates Stemness of PC3 DP Cells

**FIGURE 3:**
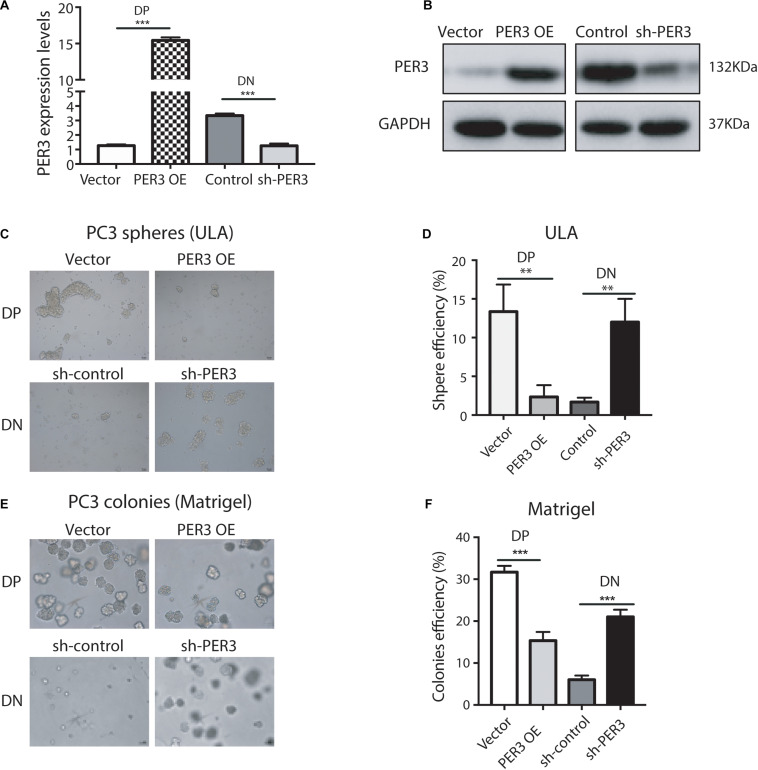
PER3 regulates stemness of PC3 DP cells *in vitro*. **(A)** RT-qPCR analysis was used to evaluate efficiency of *PER3* overexpression (OE) and knockdown (KD, or sh-PER3) in PC3 DP and PC3 DN cells, respectively. **(B)** Western blotting was performed to examine PER3 levels of PER3 OE in PC3 DP cells and PER3 KD in PC3 DN cells, respectively. **(C,D)** In sphere formation assays, *PER3* OE in PC3 cells impairs their sphere-forming abilities whereas *PER3* KD in PC3 DN cells improved their sphere formation. A total of 1,000 cells for each well were seeded in ULA plates. Spheres were imaged **(C)** and counted **(D)** for sphere efficiency 1–2 weeks later. **(E,F)** In colony formation assays, *PER3* OE in PC3 DP cells abrogates their colony-forming abilities whereas *PER3* KD in PC3 DN cells improved their clonogenicity. A total of 1,000 cells for each well were mixed with Matrigel and plated in 24-well culture plates. Colonies were imaged **(E)** and counted **(F)** for colonies efficiency 1–2 weeks later. Data are presented from three independent experiments. Scale bars, 100 μm. **P < 0.01, ***P < 0.001.

To functionally study PER3 regulation of PCSCs, we overexpressed PER3 in PC3 DP cells (Figures 3A,B) and found that PER3 overexpression (OE) in PC3 DP cells significantly inhibited their sphere-forming abilities in ultra-low attachment (ULA) plates (Figures 3C,D). In contrast, knocking down PER3 in PC3 DN cells (Figures 3A,B) led to bigger and more spheres (Figures 3C,D). Furthermore, PER3 OE in PC3 DP cells suppressed their colony-forming abilities by generating smaller and fewer colonies in Matrigel (Figures 3E,F), but PER3 KD in DN cells promoted colony formation (Figures 3E,F). Together, these data indicate that PER3 negatively regulates stem cell features of DP cells *in vitro*.

**FIGURE 4:**
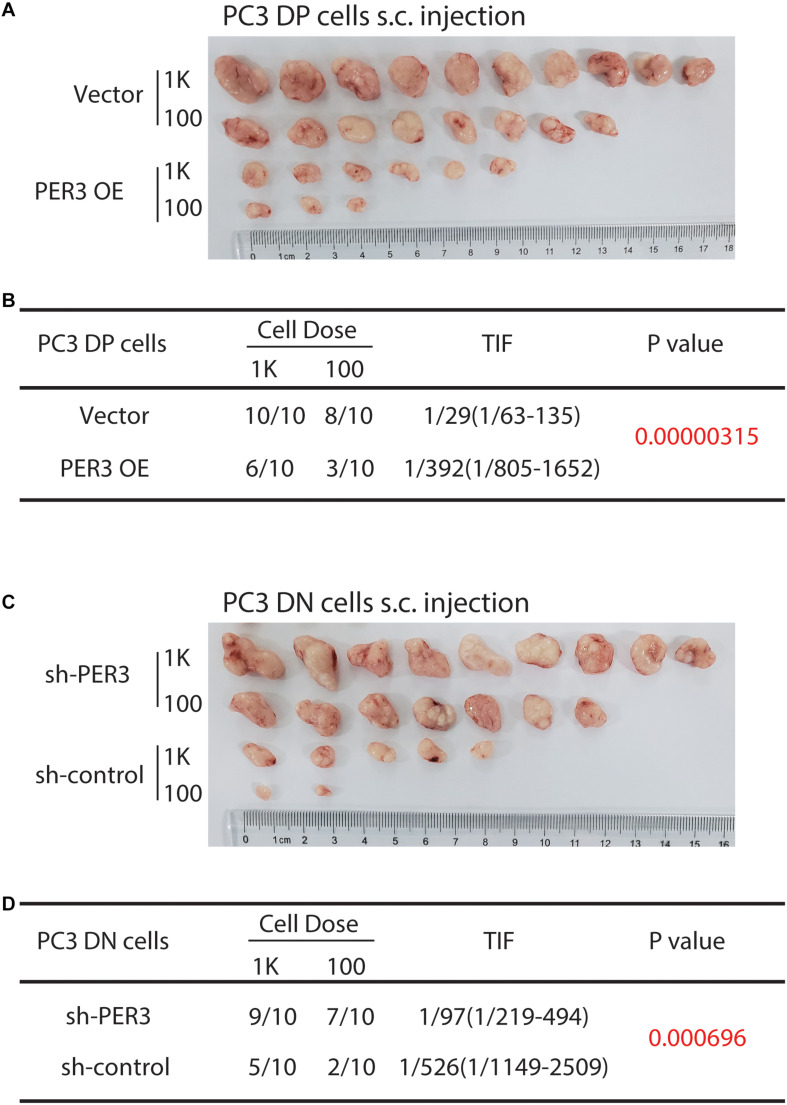
PER3 regulates stemness of PC3 DP cells *in vivo*. **(A,B)** *PER3* OE in PC3 DP cells inhibits tumor formation. DP cells freshly purified from PC3 cell line were infected with the pGMLV-PE1-PER3 lentiviral vectors (MOI = 20, 48 h) and injected subcutaneously into male NOD/SCID mice at increasing cell doses. Six weeks after implanting, tumors were harvested. Tumor images, incidence and TIF were recorded. **(C,D)** *PER3* KD in PC3 DN cells promoted their tumorigenicity. DN cells freshly purified from PC3 cell line were infected with lentiviral vectors (MOI = 20, 48 h) and subcutaneously injected into male NOD/SCID mice at increasing cell doses. Tumors were harvested after 6 weeks after injection. Tumor images, incidence and TIF were recorded.

More importantly, we attempted to test if PER3 is functionally involved in PCa development. PER3 OE in PC3 DP cells decreased tumor initiating capacities as compared to controls (TIF 1/392 vs. 1/29, respectively, *P* = 0.00000315; Figures 4A,B). However, PER3 KD in PC3 DN cells promoted tumor formation (TIF 1/97 vs. 1/526, *P* = 0.000696, Figures 4C,D). Collectively, these results indicate that PER3 is causally important for regulating SC traits.

### PER3 Is a Potentially Prognostic Marker for PCa Patients

**FIGURE 5:**
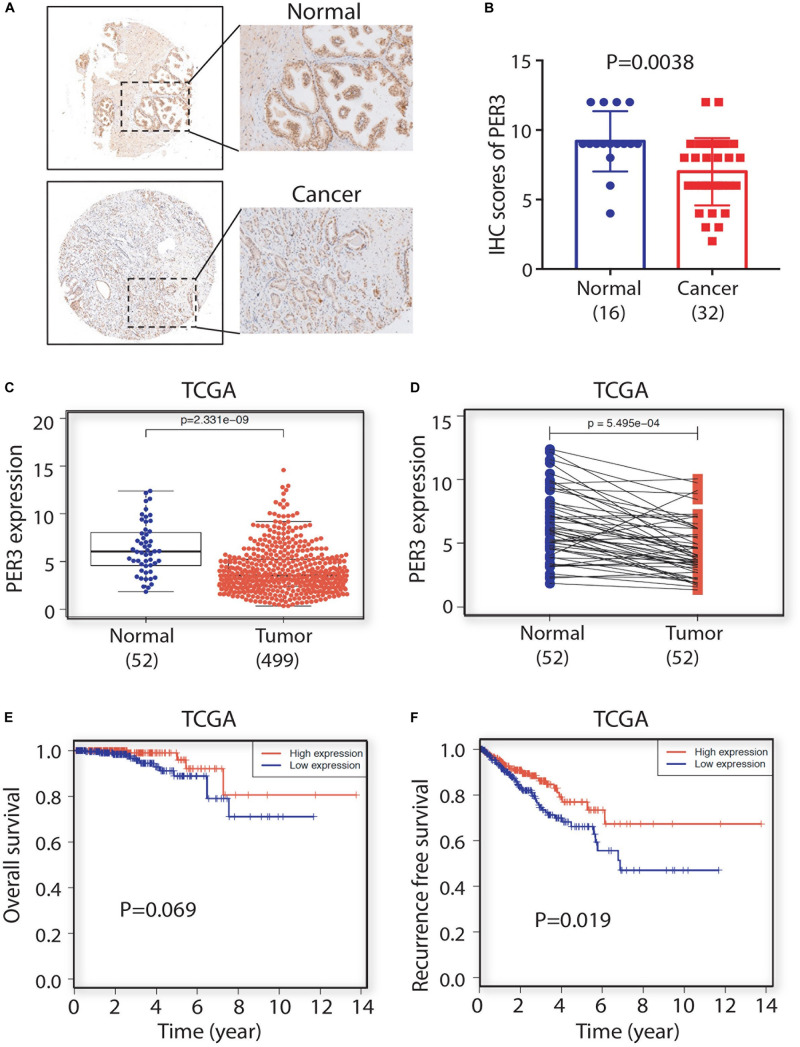
PER3 is a potentially prognostic factor for prostate cancer. **(A)** Representative IHC images of PER3 expression in benign/normal prostatic tissues and prostate cancers. **(B)** Characterizations of PER3 from a tissue microarray (TMA, Pro-01019) containing 32 untreated PCa patient and 16 normal prostate tissue cores. **(C)** *PER3* mRNA levels were downregulated in PCa in TCGA database. A total of 52 prostatic benign/normal and 499 prostate tumor tissues were compared **(C)**, and 52 matched normal and tumor pairs were compared **(D)**. **(E,F)** Kaplan-Meier analysis of the correlation of PER3 mRNA levels for overall survival rate (OS) and recurrence free survival rate in PCa patients from TCGA database.

To explore the clinical significance of PER3 in PCa, we used IHC to examine PER3 expression level in a tissue microarray (TMA) consisting of hormone naïve 32 PCa patients and 16 benign/normal prostate tissue cores. Our results revealed that PER3 levels are downregulated in HPCa tissues as compared to benign/normal tissues (Figures 5A,B). Furthermore, PCa samples in TCGA database expressed lower levels of *PER3* mRNA in HPCa tissues (*n* = 499) than normal tissues (*n* = 52) (Figures 5C,D). Moreover, the *PER3* mRNA levels are positively correlated with PCa patients’ overall survival (OS) (Figure 5E). Notably, PCa patients with higher *PER3* mRNA levels have better recurrence free survival (*P* = 0.019) (Figure 5F). Altogether, our findings suggest that PER3 has significant clinical relevance, and is a potential prognostic marker for PCa patients.

### Low Levels of PER3 Regulate Stemness of PCSCs by Activating WNT/β-Catenin Signaling

Emerging evidence has suggested that several signaling pathways play vital roles in TME for PCa development, in particular, WNT/β-catenin signaling pathways (Skvortsov et al., 2018). Surprisingly, our RNA-seq of Du145 cells revealed that the WNT/β-catenin pathway appears to be activated in PCa DP cells, but not in isogenic DN cells (Li et al., unpublished). However, it is not clear which key molecules are involved in triggering the activation of the WNT/β-catenin pathway in DP cells.

**FIGURE 6:**
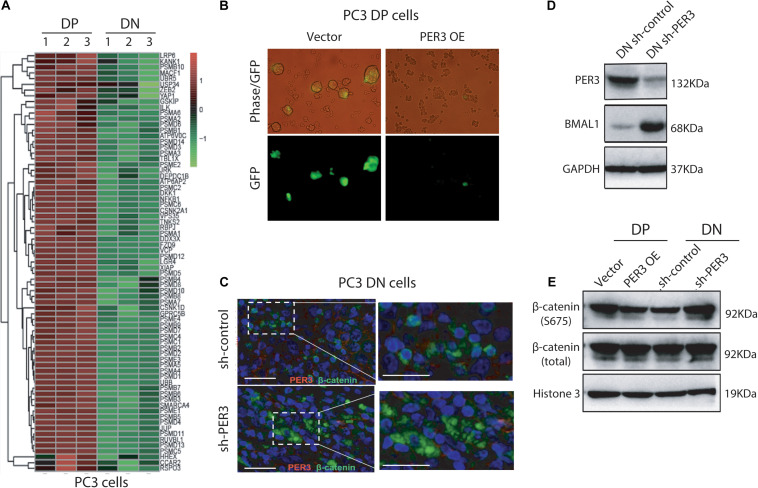
Low levels of PER3 activate WNT/β-catenin pathway. **(A)** Heatmap enrichment of genes associated with WNT/β-catenin signaling pathway in PC3 DP cells and PC3 DN cells. **(B)** Representative images showed TOP-GFP expression in PC3 DP cells transfected with the TOP-GFP lentivirus vector. Scale bars, 100 μm. **(C)** Immunofluorescent staining of PER3 and β-catenin in PC3 DN cells after PER3 KD revealed that PER3 KD promoted translocation of β-catenin from cytoplasm into nucleus. Scale bars, left: 100 μm, right: 200 μm. **(D)** PER3 KD in PC3 DN cells led to the upregulation of BMAL1. Shown are western blotting of the molecules indicated. GAPDH was used as the control. **(E)** PER3 OE in PC3 DP cells resulted in the downregulation of phosphorylated β-catenin expression level, but PER3 KD in PC3 DN cells upregulated levels of phosphorylated β-catenin. GAPDH was used as the loading control at the western blotting.

To solve this issue, we first performed GO (Gene Ontology) enrichment analysis on the PC3 DP versus DN cells, and found that WNT/β-catenin pathway related genes are significantly enriched in PC3 DP cells (Figure 6A), further supporting our observations that the WNT/β-catenin pathway may be involved in the regulation of SC features of PCa DP cells. Furthermore, we employed a TOP-GFP viral vector, a TCF/LEF reporter, which has been widely used to evaluate WNT activity (Vermeulen et al., 2010). Notably, PER3 OE in PC3 DP cells decreased GFP expression, indicating the inactivation of WNT/β-catenin pathway, whereas PER3 KD in PC3 DN cells increased GFP expression (Figure 6B; data not shown). More importantly, our results revealed that PER3 KD in PC3 DN cells led to the translocation of cytoplasmic β-catenin into the nucleus (Figure 6C), indicating that the activation of WNT/β-catenin signaling pathway. In addition, PER3 KD in PC3 DN cells resulted in the upregulation of BMAL1, which is a transcriptional activator for circadian clock genes and related to the WNT/β-catenin signaling pathway from earlier reports (Lecarpentier et al., 2019) (Figure 6D). On the other hand, PER3 OE in PC3 DP cells led to the downregulation of BMAL1 (data not shown). Finally, PER3 KD in PC3 DN cells resulted in the phosphorylation of β-catenin, whereas PER3 OE in PC3 DP cells downregulated the level of phosphorylated β-catenin (Figure 6E). Taken together, these data suggest that PER3 negatively regulates stemness of PCSCs, probably via WNT/β-catenin signaling pathway.

## Discussion

Like many solid tumors, prostate cancer cells are heterogeneous (Tang, 2012), and this cancer cell heterogeneity can be explained by both clonal evolution and/or CSC models (Lytle et al., 2018; Prager et al., 2019). In prostate cancer, emerging evidence has shown the existence of PCSCs, which are important for prostate cancer development at every stage (Deng and Tang, 2015). Through both *in vivo* and *in vitro* methods, PCSCs have also been identified in numerous studies (Skvortsov et al., 2018). For instance, human prostate tumor cells with CD44^+^α2β1^hi^CD133^+^ phenotype represent potential PCSCs *in vitro* (Collins et al., 2005). Using PCa xenograft tumors, CD44^+^ PCa cells are shown to have stem-like cancer cell properties (Patrawala et al., 2006), and CD44^+^α2β1^+^ PCa cells are further enriched in tumor-initiating cells (Patrawala et al., 2007). In addition, PSA^–/lo^ PCa cells are reported to serially propagate tumor regeneration and are resistant to androgen deprivation therapy (Qin et al., 2012). Moreover, PCa cells bearing high levels of aldehyde dehydrogenase (ALDH) activity are enriched in tumor-initiating and metastasis-imitating cells (van den Hoogen et al., 2010). Furthermore, PCSCs are reported to be responsible for therapy resistance and castration resistant prostate cancer (CRPC) (Domingo-Domenech et al., 2012; Chen et al., 2016). Recently, we have found that hormone naïve HPCa cells with ALDH^hi^CD44^+^ (DP) phenotype can give rise to bigger and more colonies or spheres than HPCa cells with ALDH^lo^CD44^–^ (DN) phenotype (Chen et al., 2016), hinting that these cells may be the cellular target for PCa treatment. To further explore this possibility, we have shown that PC3 DP cells can self-renew, and are more clonogenic and tumorigenic than the corresponding DN cells (Figure 1), consistent to our previous observations that DP cells in Du145 cell model also bear significantly enhanced metastatic potential, clonogenicity and tumorigenicity than isogenic Du145 DN cells (data not shown). These data together suggest that PCa DP cells are a subpopulation of PCSCs.

Recent evidence has suggested that the circadian clock is associated with TME in solid tumors with clinical values and therapeutic potentials, although the underlying mechanisms are not clear. For example, Zhou et al. (2020) found that a wide range of circadian clock genes are changed epigenetically in kidney renal clear cell carcinoma (KIRC), and have a prognostic value. KIRC patients expressing high levels of *PER2*, *PER3*, *CLOCK*, *CRY2*, and *RORA* have a better overall survival and disease-free survival (Zhou et al., 2020). In addition, circadian clock genes are implicated in various signaling pathways, such as apoptosis and cell cycle, as well as in immune cell infiltration (Zhou et al., 2020). Similarly, by multi-omics computation techniques, several core circadian clock genes are found changed epigenetically in lung adenocarcinomas and lung squamous cell carcinomas, which are involved in cell cycle and apoptosis, such as *PER2* and *RORA* (Yang et al., 2019). By applying MC38, a colorectal cancer cell line, in a syngeneic mouse model of liver metastasis, *Per1*^–/–^*Per2*^–/–^ mice had reduced liver metastasis, and *Per2*^–/–^ livers had less cancer-associated fibroblasts infiltration and collagen deposition (Shaashua et al., 2020). Further characterizations revealed that stromal *Per2* is also required for primary tumor formation (Shaashua et al., 2020). This finding implies the importance of PER family genes in TME.

How circadian rhythm genes regulate CSCs remains unknown. In a mouse model of acute myeloid leukemia (AML), *in vivo* RNAi screening identified that the circadian rhythm genes (Bmal1 and Clock) are required for AML cell growth, and targeting the circadian machinery showed therapeutic effects by depleting leukemia stem cells and impaired cell proliferation (Puram et al., 2016). In solid tumors, glioblastoma stem cells (GSCs) dependent on core circadian clock transcription factors, BMAL1 and CLOCK, for their cell growth, and targeting core clock factors in GSCs suppresses their tumor growth (Dong et al., 2019). Moreover, Bmal1 KO reduced the development of murine skin tumors by reducing tumor-initiating cells and enhancing the expression of tumor suppressor genes (Janich et al., 2011). These studies, together with many others, have highlighted the impact of circadian clock genes on CSCs.

**FIGURE 7:**
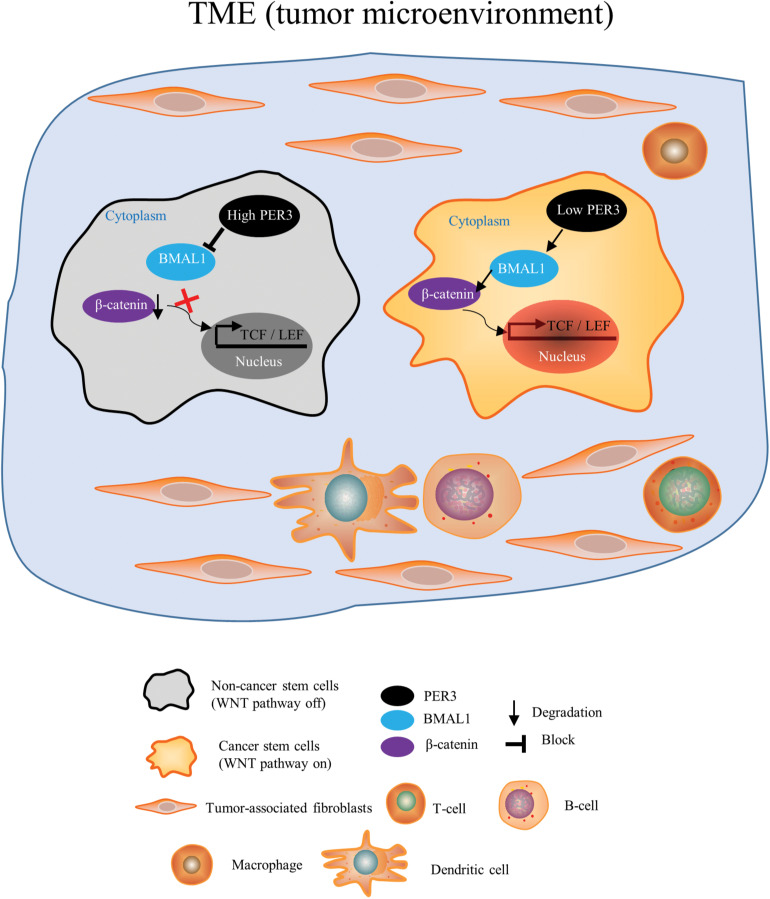
A model depicting that PER3 regulates stemness of PCSCs via WNT/β-catenin pathway. Our results suggest that in PCa TME, PCa DN cells (non-PCSCs) bear high levels of PER3, which downregulates the expression of BMAL1 and prevents the translocation of β-catenin from cytoplasm into nucleus to activate WNT/β-catenin pathway. On the other hand, PCa DP cells (PCSCs) have low levels of PER3, which upregulates BMAL1 expression, leading to the translocation of β-catenin from cytoplasm into nucleus to eventually activate WNT/β-catenin signaling pathway.

In this study, we are the first to report the biological effect of circadian clock genes on PCSCs. We have made several novel findings. First, using deep RNA-Seq and applying systematic bioinformatic analysis, we found that the circadian rhythm pathway is significantly downregulated in PC3 DP cells (vs. PC3 DN cells). Among PER family members, PER3 is markedly downregulated in PC3 DP cells, suggesting that circadian clock genes may regulate PCSCs. Second, RNA-Seq in PC3 DP cells (compared to PC3 DN cells) also revealed that PC3 DP cells are enriched in stem cell pathways (including KRAS, JAK/STAT3, and STAT5 signaling pathways), inflammatory response and interferon response (Figure 2E; Supplementary Figure 2). In support, our recent RNA-Seq in Du145 DP cells (vs. isogenic DN cells) identified that Du145 DP cells are enriched in EMT, angiogenesis, metabolisms and stem cell signaling pathways (including human embryonic stem cell pluripotency, STAT3, WNT/β-catenin signaling pathways) (Li et al., unpublished). These data suggest that PCa DP cells play vital roles in PCa TME. Third, we explored if PER3 is a key molecule to regulate PCSCs through detailed *in vitro* and *in vivo* characterizations. In colony-formation and sphere-formation assays, overexpressing PER3 in PC3 DP cells significantly suppresses clonogenicity by generating smaller and fewer colonies and/or spheres, while knocking down PER3 in PC3 DN cells markedly improves their clonogenicity. In animal studies, PER3 OE dramatically inhibits tumor initiation of PC3 DP cells, whereas PER3 KD in PC3 DN cells notably promotes their tumorigenicity (Figure 3 and Figure 4). These results altogether indicate that PER3 regulates stem cell characteristics of PCSCs. Fourth, patients with higher levels of PER3 have a better survival and disease-free rate, implying the prognostic value of PER3 in PCa. In other solid tumors, low levels of PER3 are identified in colon cancers vs. normal tissues, which are associated with colon cancer incidence and development (Wang et al., 2012). Further studies revealed that PER3 may be important in regulating the stemness of colorectal CSCs by inhibiting NOTCH and β-catenin signaling (Zhang et al., 2017), supporting our findings that the importance of PER3 in regulating PCSCs. Moreover, a study from Cai et al. (2018) reported that PER3 expression is lower in paclitaxel-resistant PCa cells, and PER3 OE in PCa resistant cells inhibits cell proliferation, arrests the cell cycle and increases apoptosis, which induce therapeutic sensitivity to paclitaxel by downregulating NOTCH1 signaling pathway, further supporting our suggestion that PER3 is of clinical and therapeutic value. Mechanistically, our GO enrichment analysis identified that many genes related to WNT/β-catenin pathway are significantly enriched in PC3 DP cells, as WNT/β-catenin signaling pathways have been shown to be imperative for PCSC regulation (Skvortsov et al., 2018). More importantly, PER3 KD in PC3 DN cells increases the expression of BMAL1, resulting in the phosphorylation of β-catenin and translocation of β-catenin from the cytoplasm into the nucleus to activate the WNT/β-catenin pathway (Figure 7). On the contrary, PER3 OE in PC3 DP cells inhibits the expression of BMAL1, leading to the inactivation of the WNT/β-catenin pathway (Figure 7). The exact mechanisms of how PER3 regulates stemness of PCSCs via WNT/β-catenin pathway will be further clarified.

Circadian clock dysfunction may play a role in cancer development, and this relationship may be instrumental in the development of targeted treatments for cancer patients (Sancar and Van Gelder, 2021). Our ongoing work is to elucidate the exact mechanisms of the circadian clock genes (PER3) on PCSCs, which hopefully can be translated into clinical management in the future.

## Data Availability Statement

The datasets presented in this study can be found in online repositories. The names of the repository/repositories and accession number(s) can be found below: The RNA-Seq data presented in the study are deposited in the Sequence Read Archive (SRA) (www.ncbi.nlm.nih.gov/sra/), Accession number: PRJNA671757.

## Ethics Statement

The animal study was reviewed and approved by the IACUC of Huazhong University of Science and Technology.

## Author Contributions

QLL, QHL, and XC designed all the experiments and the concept of this manuscript. QLL performed the major experiments. DX, ZHW, BL, JZ, PP, QJT, JD, JG, and DK provided technical support. WMC and JM provided scientific suggestions. QLL and XC drafted the manuscript. JM and XC co-supervised QLL to complete this project. All authors listed have read and approved the manuscript before submission.

## Conflict of Interest

The authors declare that the research was conducted in the absence of any commercial or financial relationships that could be construed as a potential conflict of interest.

## Footnotes

1 http://bioinf.wehi.edu.au/software/elda/

2 ftp://ftp.ensembl.org/pub/, GRCh38.p12

3 www.ncbi.nlm.nih.gov/sra/

## Supplementary Material

Supplementary Figure 1 Bioinformatic analysis of RNA-Seq in PC3 DP cells compared to PC3 DN cells. **(A)** High consistency in the biological triplicates were shown in the correlation plot. **(B)** Proper distribution of fpkm of each analyzed sample was shown in the violin plot.

Supplementary Figure 2 GSEA shows enrichment of gene signatures in PC3 DP cells compared to DN cells in TME. **(A)** GSEA shows enrichment of gene signatures related to CSCs, including IL-2/STAT5 pathway. **(B)** GSEA shows enrichment of gene signatures pertinent to inflammatory response.

## Abbreviations

ALDH: aldehyde dehydrogenase
AML: acute myeloid leukemia
CSCs: cancer stem cells
DN cells: ALDH^lo^CD44^–^ cells
DP cells: ALDH^hi^CD44^+^ cells
FACS: fluorescence-activated cell sorting
GSCs: glioblastoma stem cells
IHC: immunohistochemical staining
KIRC: kidney renal clear cell carcinoma
LDAs: limiting dilution assays
mCRPC: metastatic castration resistant prostate cancer
OS: overall survival
PCa: prostate cancer
PCSCs: prostate cancer stem cells
PER3: period circadian regulator 3
RNA-seq: RNA-sequencing
s.c.: subcutaneous
TIF: tumor-initiating frequency
TMA: tissue microarray
TME: tumor microenvironment
ULA: ultra-low attachment.
