# Supplementary figures and images for: Circadian Rhythm Gene PER3 Negatively Regulates Stemness of Prostate Cancer Stem Cells via WNT/β-Catenin Signaling in Tumor Microenvironment
Source: Front Cell Dev Biol. 2021 Mar 18;9:656981. doi: 10.3389/fcell.2021.656981 (PMC8012816; doi:10.3389/fcell.2021.656981)

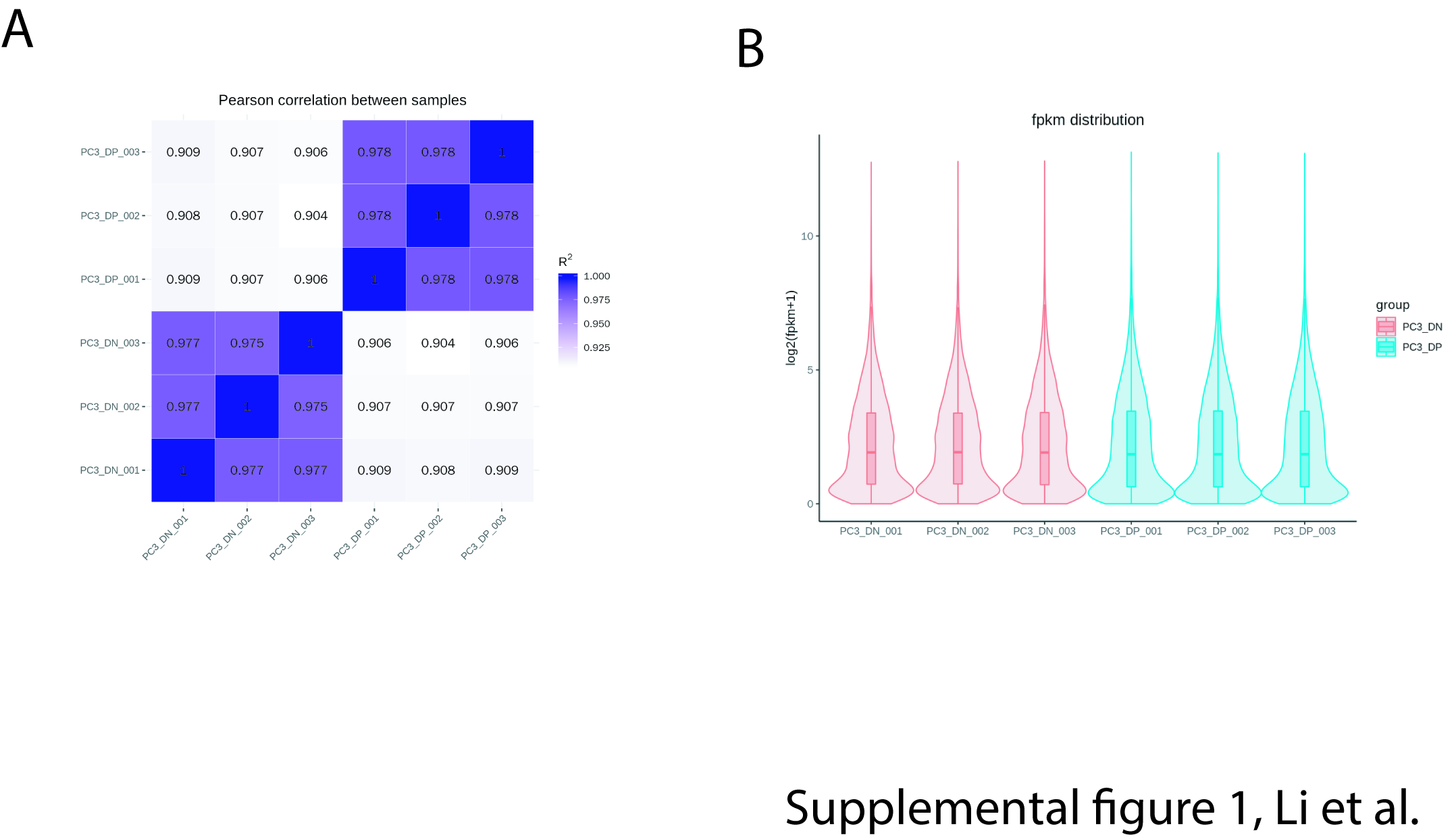

Supplement: Supplementary Figure 1 — Bioinformatic analysis of RNA-Seq in PC3 DP cells compared to PC3 DN cells. (A) High consistency in the biological triplicates were shown in the correlation plot. (B) Proper distribution of fpkm of each analyzed sample was shown in the violin plot. [file Image_1.TIF]

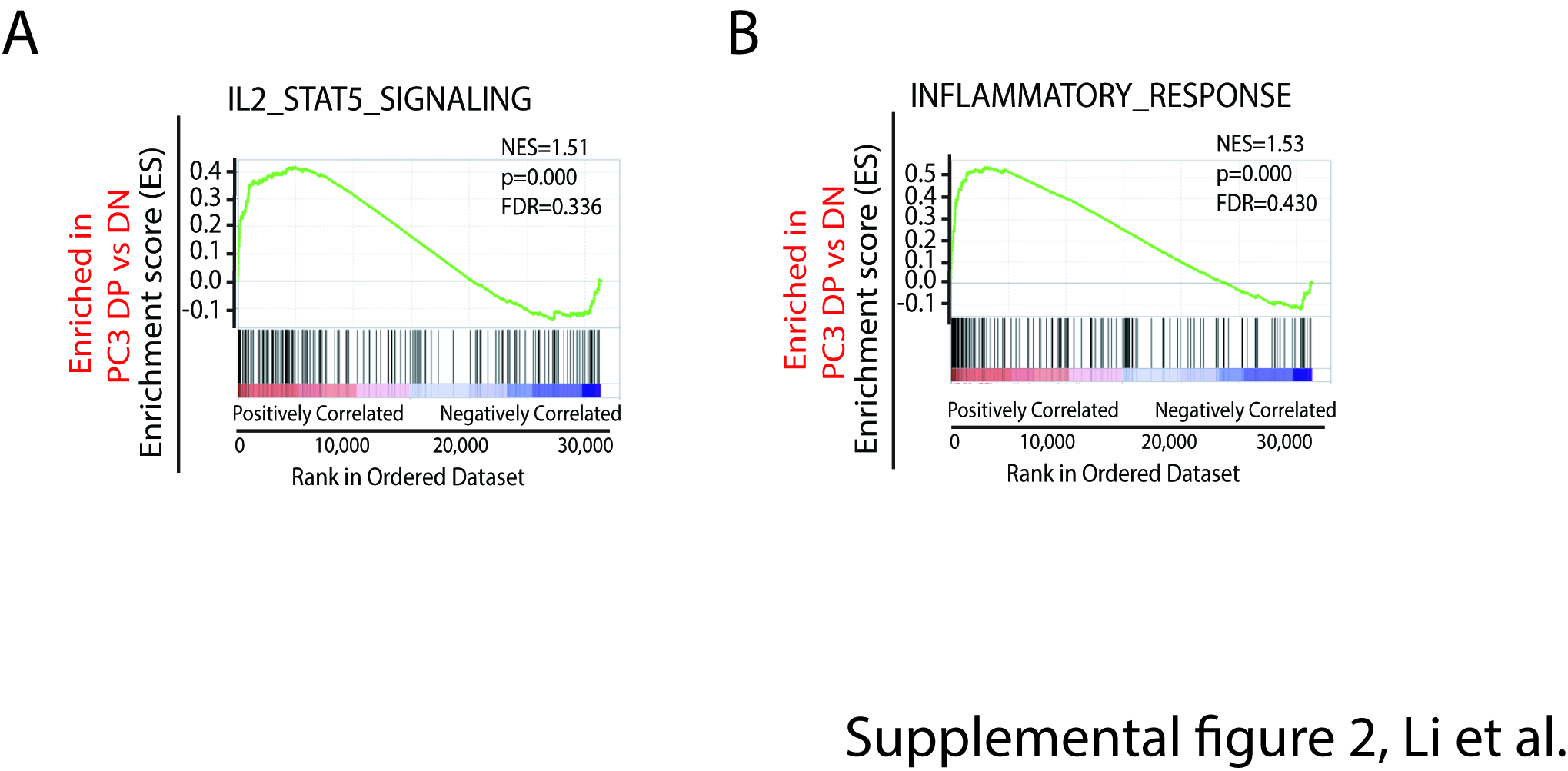

Supplement: Supplementary Figure 2 — GSEA shows enrichment of gene signatures in PC3 DP cells compared to DN cells in TME. (A) GSEA shows enrichment of gene signatures related to CSCs, including IL-2/STAT5 pathway. (B) GSEA shows enrichment of gene signatures pertinent to inflammatory response. [file Image_2.TIF]
